# Supplementary material for: From sequence to enzyme mechanism using multi-label machine learning
Source: BMC Bioinformatics. 2014 May 19;15:150. doi: 10.1186/1471-2105-15-150 (PMC4229970; doi:10.1186/1471-2105-15-150)
Supplement: Additional file 2 — Java code of ml2db. Additional file ml2db_code.tar.gz contains the Java source code to run the multi-label machine learning experiments and save the results to database. The code’s Javadoc is included. [file 1471-2105-15-150-S2.zip › additional file 2/ml2db/ml2db/doc/main/ml2db/mulan/predict/PredictionsRecordTable.html]

PredictionsRecordTable


JavaScript is disabled on your browser.


- Overview
- Package
- Class
- Use
- Tree
- Deprecated
- Index
- Help

- Prev Class
- Next Class

- Frames
- No Frames

- All Classes

- Summary:
- Nested |
- Field |
- Constr |
- Method

- Detail:
- Field |
- Constr |
- Method


main.ml2db.mulan.predict

## Class PredictionsRecordTable

- java.lang.Object
- - uk.ac.standrews.utils.main.database.Table
  - - main.ml2db.mulan.predict.PredictionsRecordTable

- ---

    

  ```
  public class PredictionsRecordTable
  extends uk.ac.standrews.utils.main.database.Table
  ```

  The table containing the predictions (experiment identifier, instance, label,
  confidence etc.) for a prediction experiment. The experiment this data refers
  to (a set containing an experiment identifier, algorithm, date etc. ) is
  stored in a separate table.

  Version:
  :   21 Mar 2013

  Author:
  :   ldf

- - ### Field Summary

    Fields

    | Modifier and Type | Field and Description |
    | --- | --- |
    | `static java.lang.String` | `CONFIDENCE` the confidence in the prediction, where available |
    | `static java.lang.String` | `EXPERIMENT_ID` the identifier of the prediction experiment (algorithm, data, start time etc.) this record belongs to |
    | `static java.lang.String` | `INSTANCE` |
    | `static java.lang.String` | `LABEL` |
    | `static boolean` | `m_debug` |
    | `static java.lang.String` | `PRED_RECORDS_TABLE_NAME` |
    | `static java.lang.String` | `STATUS` the prediction status compared with the true label: true positive, false positive etc. |

    - ### Fields inherited from class uk.ac.standrews.utils.main.database.Table

      `m_writer`
  - ### Constructor Summary

    Constructors

    Constructor and Description

    `PredictionsRecordTable(uk.ac.standrews.utils.main.database.DbManager dbManager)`
  - ### Method Summary

    Methods

    | Modifier and Type | Method and Description |
    | --- | --- |
    | `static java.util.Vector<uk.ac.standrews.utils.main.database.TableColumn>` | `getColumnDefinitions()` |
    | `void` | `saveRowPreparedStatement(int experimentId, java.lang.String[] instanceIdentifier, java.lang.String[] labelName, double[] confidence, java.lang.String[] status)` Save using a prepared statement in small batches. |

    - ### Methods inherited from class uk.ac.standrews.utils.main.database.Table

      `getColumnNames, getColumns, getDbManager, getTableCreator, getTableName, getTableReader, getTableRow, getTableWriter, hasKey, initialise, initialise, name, saveRow`
    - ### Methods inherited from class java.lang.Object

      `equals, getClass, hashCode, notify, notifyAll, toString, wait, wait, wait`

- - ### Field Detail


    - #### EXPERIMENT\_ID

      ```
      public static final java.lang.String EXPERIMENT_ID
      ```

      the identifier of the prediction experiment (algorithm, data, start time
      etc.) this record belongs to

      See Also:
      :   Constant Field Values


    - #### PRED\_RECORDS\_TABLE\_NAME

      ```
      public static java.lang.String PRED_RECORDS_TABLE_NAME
      ```


    - #### INSTANCE

      ```
      public static java.lang.String INSTANCE
      ```


    - #### LABEL

      ```
      public static java.lang.String LABEL
      ```


    - #### CONFIDENCE

      ```
      public static java.lang.String CONFIDENCE
      ```

      the confidence in the prediction, where available


    - #### STATUS

      ```
      public static java.lang.String STATUS
      ```

      the prediction status compared with the true label: true positive, false
      positive etc.


    - #### m\_debug

      ```
      public static boolean m_debug
      ```
  - ### Constructor Detail


    - #### PredictionsRecordTable

      ```
      public PredictionsRecordTable(uk.ac.standrews.utils.main.database.DbManager dbManager)
      ```

      Parameters:
      :   `dbManager` -
  - ### Method Detail


    - #### saveRowPreparedStatement

      ```
      public void saveRowPreparedStatement(int experimentId,
                                  java.lang.String[] instanceIdentifier,
                                  java.lang.String[] labelName,
                                  double[] confidence,
                                  java.lang.String[] status)
      ```

      Save using a prepared statement in small batches.

      Parameters:
      :   `experimentId` -
      :   `instanceIdentifier` -
      :   `labelName` -
      :   `confidence` -
      :   `status` -


    - #### getColumnDefinitions

      ```
      public static java.util.Vector<uk.ac.standrews.utils.main.database.TableColumn> getColumnDefinitions()
      ```


- Overview
- Package
- Class
- Use
- Tree
- Deprecated
- Index
- Help

- Prev Class
- Next Class

- Frames
- No Frames

- All Classes

- Summary:
- Nested |
- Field |
- Constr |
- Method

- Detail:
- Field |
- Constr |
- Method
